# Practical Points

**Published:** 1895-04-20

**Authors:** 


### PRACTICAL POINTS.

[Questions are invited for this column on any point in the administration of hospitals and asylums which may prove of difficulty or interest to our readers. All communications should be marked "Practical Points," addressed to the Editor of The Hospital, 428, Strand, W.C.]

**Cavity Walls for Hospital Wards.**—Architect writes: *I shall be much obliged if you will favour me with your opinion as to the desirability or otherwise of cavity walls for hospital wards. Your book on hospitals is silent on this point, and several hospitals which I have seen have apparently solid walls, but a brick wall 18 in. in thickness will not keep out drifting rain. I have seen it come through a 2-ft. wall of Dartmoor granite on the moorside.*

If these are necessary by reason of the porousness of local materials used for walling, or the extremely exposed position of the building, the utmost care should be taken to build the inner skin thick enough to secure the cavity against any connection with the interior of the hospital. The inner wall should never be less than nine inches thick, if brick is used, or the equivalent in other materials, otherwise, the fixing of the joinery, floor joists, &c., may initiate a communication between the hospital wards and the cavity, which then becomes a harbour for vermin and a breeding place for disease germs. Solid walls are best, where circumstances do not compel the use of cavity walls, though the latter, when properly constructed, conduce to dryness and equality of temperature.

